# Advances in Eco-friendly Materials for Sustainable Packaging and Single-Use Utensils: A Decade of Innovation in Preparation, Characterization, and Applications

**DOI:** 10.1021/acsami.5c16814

**Published:** 2025-10-16

**Authors:** Guilherme Jose Aguilar, Alan Maicon de Oliveira, Pedro Esteves Duarte Augusto, Delia Rita Tapia-Blácido

**Affiliations:** † Department of Chemistry, Faculty of Philosophy, Science and Letters at Ribeirão Preto, 28133 University of São Paulo, 14040-901 Ribeirão Preto, São Paulo, Brazil; ‡ Ribeirão Preto College of Nursing, University of São Paulo, 14040-901 Ribeirão Preto, São Paulo, Brazil; § Université Paris-Saclay, CentraleSupélec, Laboratoire de Génie des Procédés et Matériaux, Centre Européen de Biotechnologie et de Bioéconomie (CEBB), 3 rue des Rouges Terres, 51110 Pomacle, France

**Keywords:** Sustainable, Film, Foam, Glycerol, Active, Packaging

## Abstract

The demand for sustainable alternatives to synthetic plastics increases, driving research on developing eco-friendly packaging and single-use utensils. Unlike previous reviews, this work provides a critical synthesis of technological trends, identifying gaps that need to be filled and highlighting innovations that are reshaping the field, considering the scientific studies in the past decade. For this, we analyzed 5,744 articles published between 2013 and 2024, selected from 26,080 records retrieved in the Scopus database, through a scoping review aligned with the PRISMA-ScR checklist. Our findings reveal that research has been largely concentrated on films (over 90%), underscoring the need to develop other material types such as foams, cups, straws, and trays. Starch, cellulose, and poly­(lactic acid) were the main raw materials with glycerol as the predominant plasticizer. Cross-linkers, fillers, and nanomaterials were incorporated to enhance mechanical, barrier, and functional properties—even though some of them are not biobased nor sustainable. Vegetable extracts and essential oils were used to impart active properties. Evaluations of biodegradability or disintegration, antimicrobial activity, and food preservation applications increased significantly between 2019–2024 compared to 2013–2018, demonstrating a shift toward multifunctional, application-oriented solutions and a growing demand for bioactive packaging. Overall, this review contributes by providing a comprehensive landscape of the field, highlighting the transition toward multifunctional, biodegradable, and innovative packages and utensils while emphasizing the challenges that must be overcome for broader industrial application.

## Graphical abstract

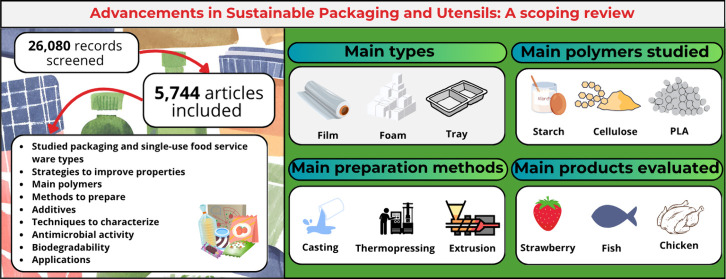

## 1 Introduction

Growing environmental awareness and the urgent need to mitigate the impacts of climate change have transformed various industrial sectors, including packaging. Packaging is defined as a container or wrapping that temporarily stores products, either individually or in groups, with the primary function of protecting them and extending their shelf life.^1^ Packaging has become fundamental in modern society, being essential for food supply, health, and well-being while also improving consumption, product identification, and distribution.

Nevertheless, this sector is a major source of solid waste and petroleum utilization, which leads to severe environmental consequences such as plastic pollution and depleted natural resources, particularly when petroleum-based derivatives are concerned.^2,3^ Materials developed to be used as a temporary utensil, such as single-use utensils (trays, bags, cups, sachets, tableware, cutlery, plates, boxes, containers, and bowls), present the same problem.

In this context, developing biobased eco-friendly sustainable packaging and single-use utensils has become a global priority, fueled by scientific advancements, regulatory changes, and increasing pressure from consumers who are more aware of their environmental footprint. However, public perception and knowledge of sustainable packaging options determine whether consumers adopt them. Indeed, consumer behavior is influenced by how consumers understand environmental issues and perceive the benefits of sustainable alternatives. For instance, if consumers are not properly informed about how conventional plastics, biodegradable packaging, and compostable options differ, they may struggle to make choices aligned with sustainability goals.^4–6^ Sustainability in packaging can be defined as an optimized, quantified, and validated approach that balances social, economic, ecological, and safety aspects across the entire circular value chain, considering the full life cycle of the food product–package unit. This definition highlights that sustainability involves not only material selection or biodegradability but also production processes, functional performance, end-of-life management, and overall environmental and societal impacts.^7^

Over the past decades, the sustainable packaging sector has advanced, driven primarily by the adoption of renewable raw materials. Nevertheless, challenges persist, including high production costs, competition for land that could be used for more critical applications like food production, and the need to balance environmental sustainability and functional performance.^8,9^ Additionally, global impact assessments have revealed that biobased packaging may have a high environmental footprint, especially when established infrastructure and resource efficiency are considered.^10^ To address these issues, researchers and industry stakeholders have been exploring innovative strategies, such as reducing the use of pure polymers by blending them with waste materials or agricultural byproducts, thereby minimizing resource consumption and environmental impact.^11,12^

To overcome these challenges, researchers have employed diverse strategies to prepare biobased materials, including developing matrixes consisting of polymers derived from natural sources, such as poly­(lactic acid),^13^ starch,^14,15^ gelatin,^16^ soybean protein,^17^ chitosan,^18^ poly­(butylene adipate-*co*-terephthalate),^19^ and cellulose;^20^ incorporating functional additives like antimicrobial agents into polymer matrixes;^18,19^ modifying the polymer structure to improve packaging properties (e.g., adding cross-linkers to polymer matrixes;^21,22^ ozonation;^23^ dry heat treatment^24^) and enhancing packaging biodegradability (e.g., blending matrixes with biodegradable polymers^25^). Furthermore, using optimized processing conditions, reusable materials, and fillers has contributed to enhancing the properties of sustainable packaging and broadening its applications.

Over the years, this multifaceted approach has led to the production of an array of sustainable packaging, reflecting continuous innovation and refinement. Therefore, this review aims to explore how sustainable packaging and single-use utensil technologies progressed between 2013 and 2024 and to highlight emerging trends, persistent challenges, and future opportunities. In this review, single-use utensils were included alongside packaging because both consist of disposable materials that contribute significantly to environmental impacts—being, currently, mainly produced using petroleum-based plastics. While traditional packaging primarily serves to protect, contain, and transport goods, single-use utensils are directly related to food consumption and generate substantial waste after a single use. This Perspective also aims to provide a comprehensive overview of the goals achieved in this field and to contribute to guiding future research into more effective solutions to address global environmental challenges.

## 2 Methodology

The adopted methodology, outlined by Arksey and O’Malley, adhered to the PRISMA-ScR (Preferred Reporting Items for Systematic Reviews and Meta-Analyses Extension for Scoping Reviews) checklist.^26^ The Scopus database was used, and scientific articles were published in English, Portuguese, and Spanish, covering the period from January 2013 to December 2024 (12 Years). The selection process involved establishing the eligibility criteria, developing a search strategy, selecting evidence sources, gathering data, and summarizing findings. Each stage of the methodology, documented and detailed in a protocol registered beforehand,^27^ is described in Supporting Information 1. The data were managed by employing the specialized “RAYYAN” software (SaaS), which supports systematic reviews by streamlining the screening and organization of scientific articles. This approach facilitates organization and analysis while ensuring that the review process remains transparent and reproducible.^28^ When publishing studies, authors must pay close attention to selecting the best keywords, title, and abstract as these elements are crucial for ensuring that the research is easily discoverable and accessible in the scientific literature. Proper selection of these components not only increases the visibility of the study but also facilitates its retrieval in bibliographic searches, enhancing its impact and relevance.

The selection of ideal terms is a critical step, especially in systematic reviews, as it directly influences the quality and scope of the results obtained. In the context of sustainable packaging and single-use utensils, it is essential to consider both technical terms and broader concepts, as terminology can vary significantly across different research areas. For this study, the following descriptors were used in the Scopus database: TITLE-ABS-KEY­(“produc*” OR “prepar*” OR “develop*” OR “applic*” OR “characteriz*” OR “construc*”) AND TITLE-ABS-KEY­(“package*” OR “cup” OR “cups” OR “plate” OR “plates” OR “platter” OR “tray” OR “trays” OR “cutlery” OR “knife” OR “knives” OR “spoon” OR “spoons” OR “fork” OR “forks” OR “film” OR “films” OR “foam” OR “foams”) AND TITLE-ABS-KEY­(“ecological” OR “sustainable” OR “biobased” OR “biodegradable” OR “biopackag*” OR “bio-packag*” OR “bio packag*”). This combination of terms was carefully designed to cover the main aspects related to the production, development, application, and characterization of sustainable materials for application as food packages or utensils (Table 1).

**Table 1:** Inclusion and Exclusion Criteria for the Studies

Inclusion	Exclusion
Studies describing the development of sustainable materials available on the Scopus platform.	Studies describing sustainable materials intended exclusively for other areas, such as the medical or construction sectors, were excluded.
The studies needed to explicitly mention the context, using terms such as “biobased”, “packaging”, or “sustainable” in the title, abstract, and keywords (a complete description of the terms is provided in Appendix 1).	Articles not available on the Scopus platform.
	Studies that involved biobased materials, which potentially can be used to produce packages or utensils, but did not specify a packaging application in the title, abstract, or keywords.
Primary studies (experimental articles).	Review studies, guidelines, and organizational recommendations, protocol studies, editorials, lectures, letters from editors, books, book chapters, and/or abstracts presented at conferences (even if available in Scopus platform).
Studies written in English, Spanish, and Portuguese.	Studies written in languages other than English, Spanish, or Portuguese.
Studies published between 2013 and 2024.	Studies published before 2013 or after 2024.

We acknowledge that all scoping reviews have inherent limitations, such as potential bias in study selection or the inadvertent exclusion of relevant works due to the terminology used. However, the search strategy adopted was designed to minimize these limitations, ensuring a broad and representative coverage of the literature on the topic. With this approach, we hoped to obtain robust results and contribute to a comprehensive understanding of trends and advancements in the field of sustainable packaging.

Findings were summarized using both qualitative and quantitative analyses. Data were categorized into the following themes: Studied packaging and single-use utensil types; Strategies to improve sustainable packaging and single-use utensils; Main polymers in sustainable packaging and single-use utensils; Methods to prepare sustainable packaging and single-use utensils; Additives in sustainable packaging and single-use utensils; Techniques to characterize sustainable packaging and single-use utensils; Sustainable packaging antimicrobial activity; Tests to assess sustainable packaging and single-use utensils’ biodegradability or disintegration; and Sustainable packaging applications. The percentages shown in Figures 2–4 and Tables 2–7 were calculated for the respective time intervals based on the number of articles mentioning a specific category relative to the total number of mentions within that category.

## 3 Results and Discussion

### 3.1 Screening and Number of Articles

Figure 1 illustrates the screening process adopted to select the articles included in this review and shows how the number of articles on sustainable packaging and utensils varied over 2013–2024. Of the 26,080 identified records, 5,744 articles were included in this review after evaluation (1,243 and 4,501 of the articles were published between 2013 and 2018 and between 2019 and 2024, respectively). Supporting Information 2 lists the articles considered in this review.

**Figure 1:**
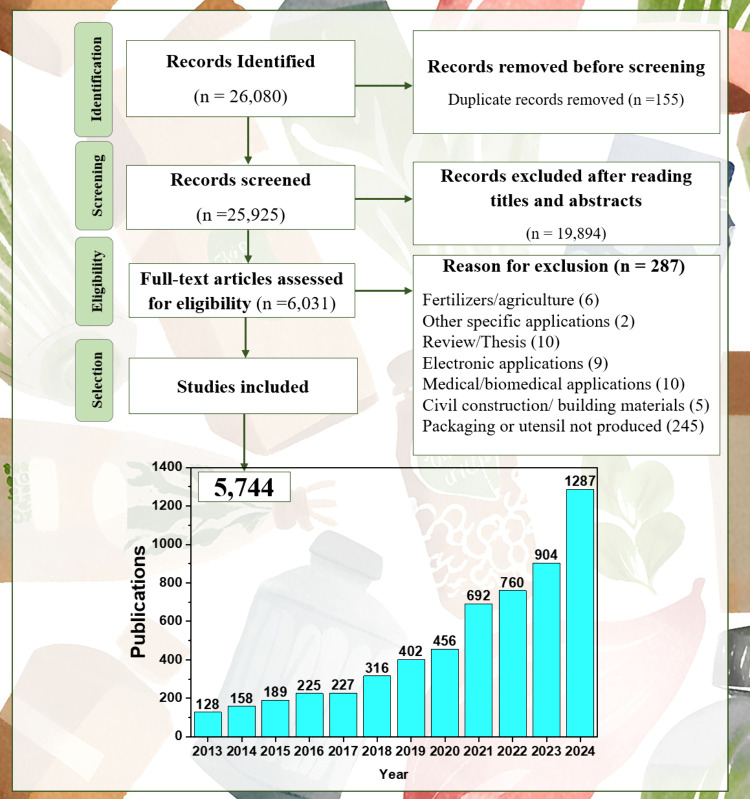
Flowchart of the article selection and inclusion process and total articles obtained in each step.

The rising number of articles from 2019 onward reflects the growing interest in sustainable materials and the increasing academic output on this subject. This aligns with heightened global awareness of environmental issues, driven by global initiatives such as the Paris Agreement (COP21) and the United Nations’ Sustainable Development Goals (SDGs).^29,30^ These initiatives have encouraged governments, industries, and researchers to fund and develop scientific studies that prioritize the search and implementation of solutions to mitigate pollution, climate change, and waste generation.^31,32^ Enforcing stricter regulations on single-use plastics and plastic waste, such as the European Single-Use Plastics Directive (2019), may also have encouraged research into innovative sustainable packaging.^33^

In addition, driven by media campaigns, documentaries, and environmental movements, consumers have become increasingly aware of how conventional plastics impact the environment, which probably underlies the growing demand for sustainable packaging and single-use utensils. Companies and brands have adopted strategies such as using the term “eco-friendly packaging” to meet consumer expectations and to improve their corporate image.^34,35^ Moreover, the COVID-19 pandemic has led consumers to rethink their use of single-use plastics. The sudden rise in demand for masks, gloves, food packaging, and medical supplies showed both the dependence on these items for safety and the growing concern about plastic pollution, highlighting the need for solutions that balance hygiene and environmental care.^11,36–38^

Thus, the rise in the number of articles regarding sustainable packaging and single-use utensils reflects not only greater acknowledgment that this topic is relevant but also a response to the social, economic, and environmental changes that have marked recent years. Combined, these factors have solidified the subject as a priority in academic and industrial agendas, suggesting that an increasing number of related studies will continue being published.

### 3.2 Studied Packaging and Single-Use Utensil Types

Figure 2 shows the sustainable materials types studied between 2013 and 2018 and between 2019 and 2024. The study period (2013–2024) was divided into six-year time intervals because this time frame should be sufficient for research and technological development to change, allowing relevant trends and advancements to be identified without the interval being so long that it dilutes important milestones. Films stood out in both time intervals: they corresponded to over 90% of the mentions of packaging and single-use utensil types. Although the percentage of mention of films decreased slightly between the first (2013–2018) and second (2019–2024) time intervals (from 93.9% to 91.7%), they remained by far the most studied packaging type, which indicates that films continue to be an area of great interest in sustainable packaging research. The slightly decreased percentage of mention of films between 2019 and 2024 reflects that the studied packaging types became diversified and points out efforts to replace non-eco-friendly packaging with more sustainable alternatives.

**Figure 2:**
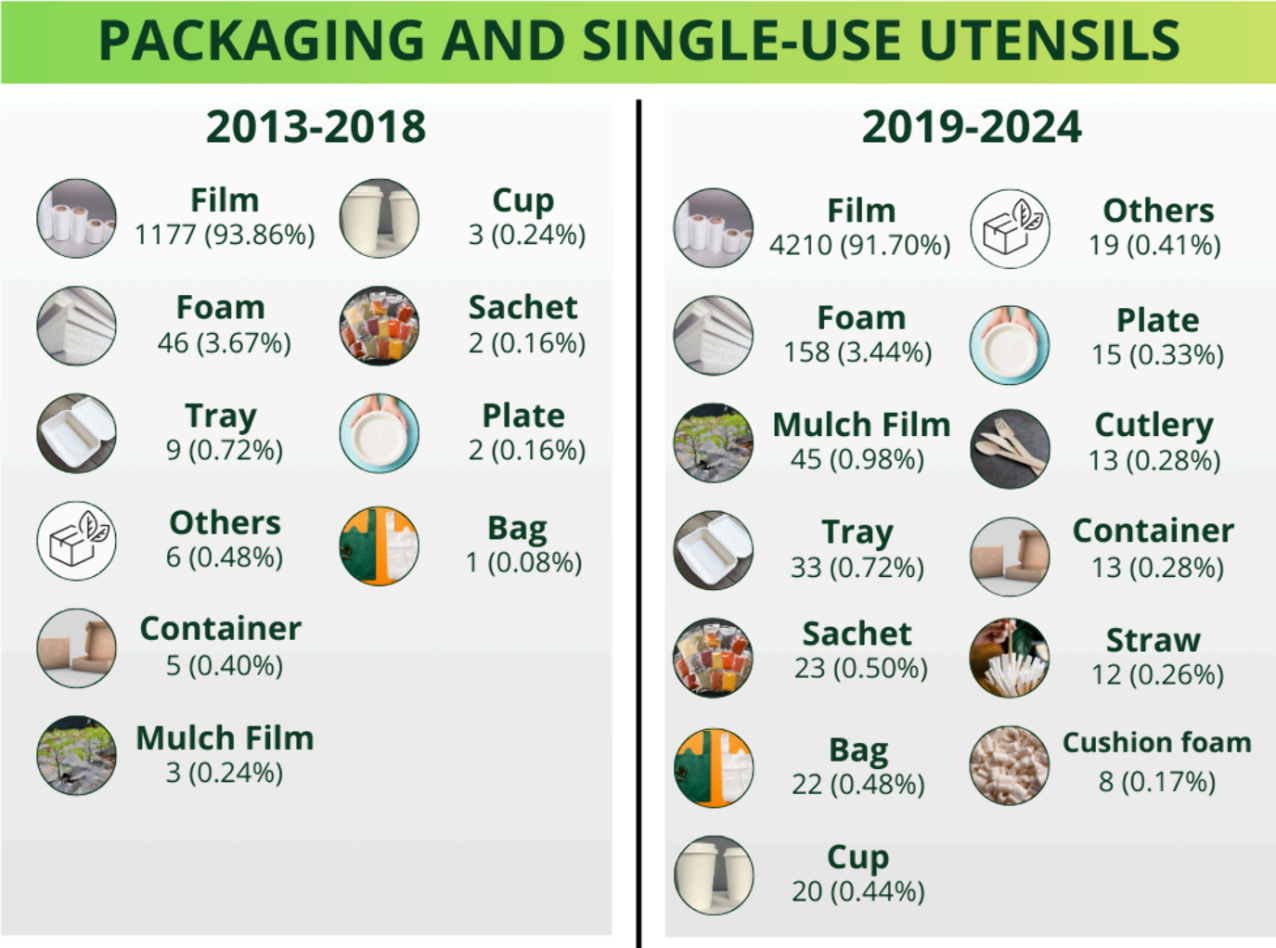
Studied packaging and single-use utensils: comparison of articles published between 2013–2018 and 2019–2024.

In 2024, the global plastic film market was valued at approximately $145 billion, and it is projected to reach $188 billion by 2029.^39^ Low-density polyethylene (LDPE), linear low-density polyethylene (LLDPE), high-density polyethylene (HDPE), and poly­(ethylene terephthalate) (PET) have dominated the plastic film segment, with LDPE accounting for over $20 billion in 2021. The Asia-Pacific region holds the largest market share, led by China, which dominates plastic film consumption in the region.^39^

Films are versatile, which supports their widespread use and allows them to be applied in various sectors, such as the agro-industrial, cosmetic, food, and healthcare areas. This extensive applicability has driven the search for more sustainable alternatives that offer mechanical and functional properties equivalent to those of conventional plastic films. This demand reflects the interest in developing packaging that combines technical efficiency and a reduced environmental impact. Moreover, they are simpler to develop in laboratories than other packages, such as rigid bottles and containers, which can also contribute to this primary choice for scientific development. Unlike packaging, which is designed to protect and transport goods, mulch films are agricultural films applied directly to soil to improve crop growth and reduce water loss.^40^ Although mulch films are not packaging nor single-use food service utensils, they are often discussed in parallel with these categories because they are also single-use plastic products with significant environmental impact.

Even so, other than films, foams, cups, trays, and straws must be developed for real applications. Compared to films, smaller volumes of foams have been produced, but this type of material plays an essential role in specific sectors such as protective packaging, thermal and acoustic insulation, and disposable products such as foam trays and cups. Moreover, foams can be used as cushion foam.^17,41–43^ This functional versatility may justify why foams were the second most studied material between 2013 and 2024.

Between 2019 and 2024, the studied materials became even more diverse, including trays, bags, cups, sachets, tableware, cutlery, plates, boxes, containers, and bowls. However, their mention remains a small percentage compared to films. Investing in studies on these types of materials could improve our understanding of their properties, expand their applications, and make them more competitive, particularly in the context of a sustainable economy. This is especially relevant for meeting the demand for environmentally friendly alternatives and diversifying the materials available in essential sectors, such as packaging and disposable products.

### 3.3 Main Polymers in Sustainable Packaging and Single-Use Utensils

Table 2 summarizes the main polymers used to produce the sustainable materials, highlighting that diverse polymers originating from biological or renewable sources were reported between 2013 and 2018 and between 2019 and 2024. Polymers extracted from plant sources, such as starch, cellulose and its derivatives, lignin, soy protein, and pectin, have been extensively studied for the development of sustainable packaging and single-use utensils.^44–46^ Algae-derived polymers include carrageenan and agar.^47,48^ Polymers of animal origin include chitosan (usually derived from chitin in crustacean shells), gelatin (derived from collagen in animal bones and skin), and whey protein (a byproduct of cheese production from milk).^49–51^ Polymers of microbial origin include poly­(lactic acid) (PLA), poly­(3-hydroxybutyrate) (PHB), poly­(3-hydroxybutyrate-3-hydroxyvalerate) (PHBV), and cellulose.^20,52,53^ Polymers prepared from synthetic or other origins include poly­(vinyl alcohol) (PVOH), polycaprolactone (PCL), poly­(butylene adipate-*co*-terephthalate) (PBAT), polyurethane, and LDPE and its derivatives. Although some of these polymers are not biodegradable or biobased, they can still be considered sustainable if produced from renewable feedstock which reduces reliance on petroleum and can lower environmental impacts across their life cycle, including carbon footprint and land-use related biodiversity effects.^54–58^ Even so, the overall use of terminology reinforced by this evaluation reinforces the need for a hierarchical classification of sustainability, which, in fact, would rarely be a definitive, unique classification, since the environmental impact is a function of each specific local context, including key factors such as sources, transport, energy sources, etc.

**Table 2:** Main Polymer-Based Packaging and Single-Use Utensil Types: Comparison of Articles Published from 2013 to 2018 and from 2019 to 2024

2013–2018		2019–2024	
Polymer	Total (%)	Polymer	Total (%)
Starch	356 (17.84)	Cellulose, its derivatives (e.g., cellulose acetate), and hemicellulose.	1172 (16.15)
Cellulose, its derivatives (e.g., cellulose acetate), and hemicellulose.	254 (12.73)	Starch	1123 (15.48)
Poly(lactic acid)—PLA	201 (10.07)	Chitosan	666 (9.18)
Chitosan	142 (7.11)	Poly(lactic acid)—PLA	546 (7.52)
Polyvinyl alcohol—PVOH	127 (6.36)	Polyvinyl alcohol—PVOH	529 (7.29)
Gelatin	95 (4.76)	Gelatin	316 (4.36)
Poly(butylene adipate-*co*-terephthalate)—PBAT	45 (2.25)	Poly(butylene adipate-*co*-terephthalate)—PBAT	313 (4.31)
Polycaprolactone—PCL	41 (2.05)	Alginate	193 (2.66)
Alginate	37 (1.85)	Lignin	173 (2.38)
Poly(3-hydroxybutyrate)—PHB	33 (1.65)	Pectin	165 (2.27)
Whey protein	33 (1.65)	Gums	146 (2.01)
Soy protein	32 (1.60)	Polycaprolactone—PCL	92 (1.27)
Lignin	31 (1.55)	Carrageenan	85 (1.17)
Polyurethane	29 (1.45)	Soy protein	77 (1.06)
Poly(3-hydroxybutyrate-3-hydroxyvalerate)—PHBV	28 (1.40)	Polyurethane	77 (1.06)
Pectin	27 (1.35)	Whey protein	71 (0.98)
Low-density polyethylene—LDPE and derivatives	25 (1.25)	Low-density polyethylene—LDPE and derivatives	68 (0.94)
Carrageenan	26 (1.30)	Poly(3-hydroxybutyrate-3-hydroxyvalerate)—PHBV	62 (0.85)
MATER-BI, Ecoflex, and other patented materials	20 (1.00)	Poly(butylene succinate)—PBS	61 (0.84)
Poly(butylene succinate)—PBS	18 (0.90)	Poly(butylene succinate-*co*-butylene adipate)—PBSA	44 (0.61)
Agar	15 (0.75)	Chitin	46 (0.63)
Other	381 (19.09)	Other	1231 (16.97)

For example, LDPE can be produced from biobased resources, such as sugar from sugar cane or CO_2_-based processes, which reduces reliance on petroleum and partially mitigates environmental impacts associated with fossil-based plastics. However, despite being derived from renewable resources, biobased LDPE is not inherently biodegradable nor compostable. Its effective use therefore requires proper end-of-life management, including reuse, recycling, and controlled disposal.^57,59,60^

Starch corresponded to 17.84% and 15.48% of the mentions of polymers between 2013 and 2018 and between 2019 and 2024, respectively, being one of the most studied polymers for sustainable materials applications. Starch is extracted from plant sources such as corn, potato, and wheat. It is a natural, biodegradable, and compostable polymer and hence an excellent choice to reduce the environmental impact of materials. However, the mechanical properties of this polymer and its interaction with water have limited its use. Indeed, films and other material types consisting solely of starch tend to be more brittle, which prevents them from being used in certain applications. To address this issue, starch has been combined with additives such as plasticizers (e.g., glycerol) to enhance its flexibility and processability.^61–64^ Similarly, Sampaio et al. (2023), in a review of 100 biobased film studies for food packaging covering the period 2010–2022, observed that gelatin, chitosan, alginate, cellulose, carrageenan, and starch were the most frequently studied polymers. This observation aligns with our data and highlights that these natural polymers remain central to research on sustainable materials due to their biodegradability, biocompatibility, and functional properties.^65^

Sustainable materials produced from cellulose and its derivatives, including carboxymethylcellulose (CMC), represent a considerable percentage of mentions between 2013 and 2018 (12.73%) and between 2019 and 2024 (16.15%). Given that cellulose is the most abundant polymer on Earth, these polymers are widely employed to obtain sustainable materials because they are biodegradable, versatile, and available. Cellulose derivatives, such as cellulose acetate, and cellulosic materials, such as paper, can be directly used for contact with products with low moisture content and water activity, or applied in multilayer materials for different applications.^45^

PLA is another key polymer. Although the percentage of mentions of PLA decreased slightly between 2019 and 2024, it continues to be a primary alternative to conventional petroleum-based plastics. PLA offers excellent transparency and mechanical strength; however, its production cost is still higher than that of traditional plastics.^10,66^ In contrast to starch and cellulose, which originate from plants, PLA originates from sugar fermentation, polymerization, and purification processes. Nonetheless, it is extensively used in sustainable food packaging and disposable products.^67,68^

Chitosan, often obtained from animal sources (derived from chitin in crustacean shells), stands out for its bioactivity, including antimicrobial and antioxidant properties, which makes it particularly suitable for the packaging of perishable food products. Considering the mention of polymers between 2013 and 2018 and between 2019 and 2024, 7.11% and 9.18% concerned chitosan, respectively. This increase reflects advancements in processing technologies and reveals that chitosan can be combined with other polymers to enhance its mechanical and barrier properties. Additionally, there is growing interest in chitin itself, as the process of deacetylating it to produce chitosan can increase production costs.^18,67,69,70^ Alginate, lignin, pectin, and protein-derived polymers, such as whey protein and gelatin, are also notable. Initially pursued to valorize abundant and low-cost byproducts, these materials have demonstrated great application potential precisely because of their advanced functionality. This reflects a dual focus: not only on sustainability through the use of undervalued resources, but also on the development of high-performance materials capable of selective barrier properties or bioactivity.^16,49,71–73^

In addition to the aforementioned polymers, Table 2 includes biobased polyurethane, polybutylene succinate, and other polymers such as plant protein derivatives. Patented polymers or commercially available formulations such as MATER-BI, a range of biobased plastics, and Ecoflex also stand out.^17,46,74,75^

Other polymers showed only marginal representation (<0.7%) in both periods. These included proteins such as gluten, zein, casein, collagen, keratin, silk fibroin, kafirin, and secalin; polysaccharides like xylan, glucomannan, galactomannan, pullulan, furcellaran, and dextrin; and less common synthetic polymers, including polyvinylpyrrolidone, poly­(propylene) carbonate, and poly­(glycolic acid).

### 3.4 Methods to Prepare Sustainable Packaging and Single-Use Utensil Types

Figure 3 presents the methods used to prepare sustainable packaging and single-use utensils. Between 2013 and 2018 and between 2019 and 2024, casting was the most used method (73.02% and 73.69%, respectively). This method is widely employed because it is efficient, simple, low-cost, and versatile, especially in the context of laboratory research into uniform and thin films.^65,76,77^ The casting method being prominent aligns with films being the most studied material type between 2013 and 2024.

**Figure 3:**
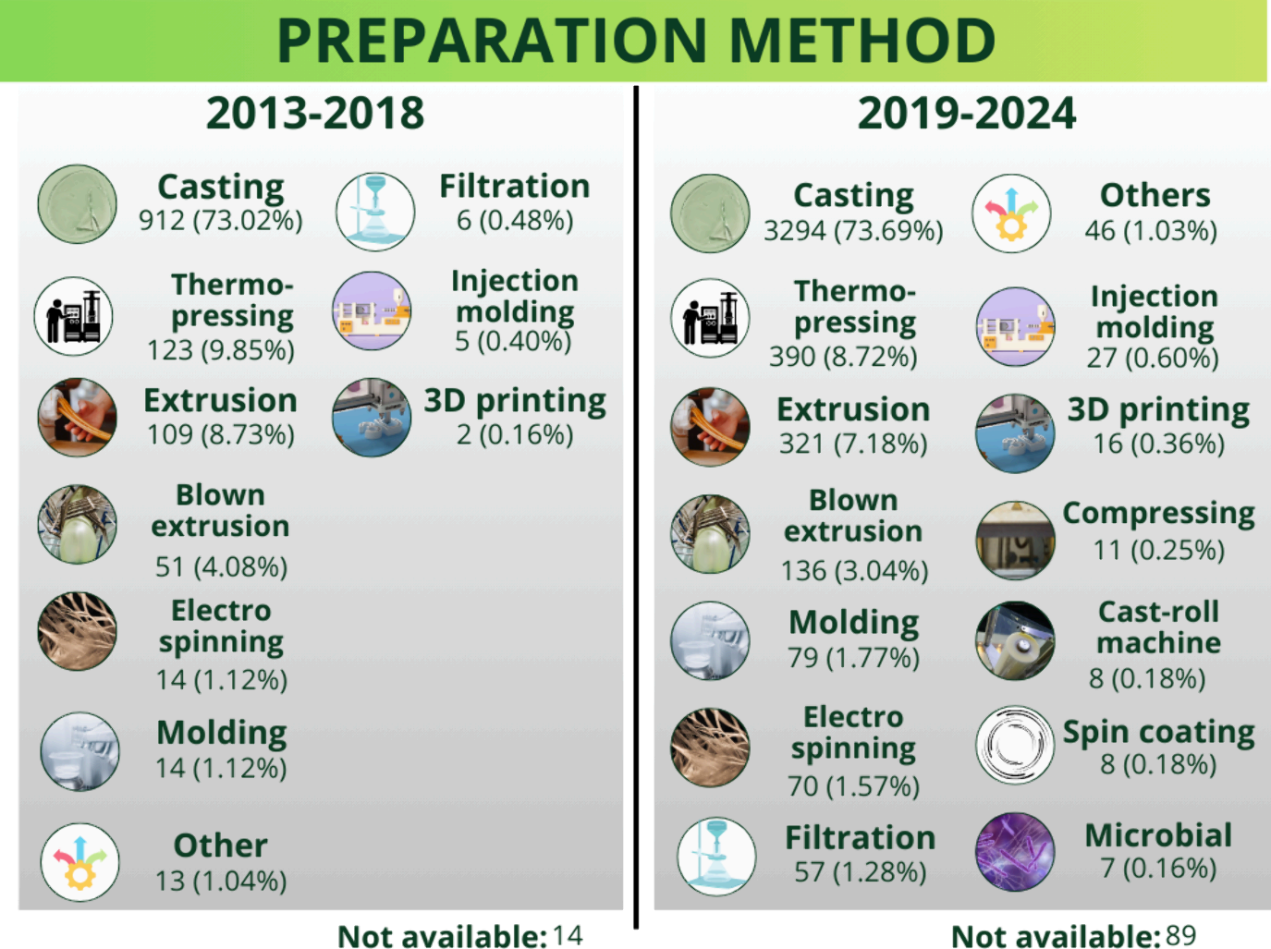
Methods to prepare sustainable packaging and single-use utensils: comparison of articles published between 2013–2018 and 2019–2024.

Other relevant methods to prepare sustainable materials include thermopressing and extrusion. Thermopressing accounted for 9.85% and 8.72% of the mentions between 2013 and 2018 and between 2019 and 2024, respectively, while extrusion represented 8.73% and 7.18% in these time intervals, respectively. Thermopressing and extrusion stand out and are employed in various industrial applications—the former is ideal for molding more rigid materials, while the latter is suitable for continuously producing films and other materials on a larger scale.^11,43,78^ Blow film extrusion is particularly noteworthy and accounted for 4.08% and 3.04% of the mentions of sustainable packaging preparation methods between 2013 and 2018 and between 2019 and 2024, respectively. This method is employed to manufacture tubular films and flexible packaging and allows films to be further molded into bags, for instance. Although widely used industrially, this technology involves relatively big and expensive equipment, limiting the studies and development in the laboratory and, particularly, scientific studies.

Although less represented, emerging methods like electrospinning and 3D printing have gained attention due to their innovative applications. Electrospinning accounted for 1.12% and 1.77% of the mentions of sustainable materials preparation methods between 2013–2018 and 2019–2024, respectively. It enables the production of ultrafine nanofibers with high surface area and tunable porosity. These nanofibrous mats can be assembled into films or coatings, which are particularly useful in multilayer packaging systems to improve barrier properties. Although scalability remains a challenge, approaches such as automated multi-injector designs and the use of nontoxic organic solvents have been proposed to enhance yield and safety.^79,80^ Meanwhile, 3D printing, with 0.16% and 0.36% of the mentions in the same periods, plays a complementary role in producing prototypes and testing experimental packaging concepts.^81,82^

Whereas research into sustainable packaging obtained by microorganism growth on premolded substrates is still limited (0.16% of the mentions of sustainable packaging preparation methods between 2019 and 2024), this method has sparked the interest of both the scientific and industrial communities.^83,84^ The process holds commercial potential, because it is sustainable and innovative. In fact, agro-industrial residues such as wheat bran, sugar cane bagasse, or sawdust can be used with minimal pretreatment, providing the growth medium for fungi. During this process, the fungal mycelium acts as a natural biobinder, colonizing the residues and forming a solid, lightweight, and biodegradable material that can be shaped into predefined structures such as trays or boxes.^85,86^ However, process limitations are still important challenges for the application of mycelium-based materials, such as the long time needed for fungal colonization and growth, the need for sterilized substrates, and post-treatments.

In conclusion, casting processes still correspond to 3/4 of the published scientific articles in the field, even though many other processing technologies are needed for real applications. Due to its simplicity and low cost, in comparison with other technologies, it is hard to indicate if casting is preferred due to its relevance or laboratory limitations or due to real-life challenges, such as grant budget and equipment costs. Even so, one important conclusion of this review is the clear necessity of expanding studies with technologies other than casting, providing materials with different characteristics for applications in both sustainable packaging and single-use utensils.

### 3.5 Additives in Sustainable Packaging and Single-Use Utensils

Table 3 summarizes the main additives used to prepare sustainable packaging. Between 2013 and 2018 and between 2019 and 2024, 17.9% and 13.9% of the articles did not use any additives, respectively, which reinforces that the majority of the produced materials need additives to achieve the desired functionality. This point is aligned with the knowledge of materials science and technology, and even traditional petroleum-based materials use additives in their production. An important and relevant point to be considered is that the additives, although applied in small quantities, can define the postusage of the produced materials (for example, limiting the recycling of traditional plastics) or compromise the classification of materials (for example, not being biobased or biodegradable).

**Table 3:** Additives Used to Prepare Sustainable Packaging and Single-Use Utensils: Comparison of Articles Published between 2013 and 2018 and between 2019 and 2024

2013–2018		2019–2024	
Additive	Total (%)	Additive	Total (%)
Glycerol	533 (29.08)	Glycerol	1920 (26.90)
Essential oil/oil (from plants)	150 (8.18)	Essential oil/oil (from plants)	576 (8.07)
Extract (from plants)	71 (3.87)	Extract (from plants)	472 (6.61)
Lignocellulosic fibers and/or particles	63 (3.44)	Lignocellulosic fibers and/or particles	300 (4.20)
Sorbitol	61 (3.33)	Zinc compounds	196 (2.75)
Montmorillonite	48 (2.62)	Citric acid	138 (1.93)
Polyethylene glycol	41 (2.24)	Sorbitol	134 (1.88)
Citric acid	33 (1.80)	Silver compounds	119 (1.67)
Zinc compounds	32 (1.75)	Tween	118 (1.65)
Tween	31 (1.69)	Polyethylene glycol	103 (1.44)
Glutaraldehyde	24 (1.31)	Calcium compounds	102 (1.43)
Titanium compounds	24 (1.31)	Montmorillonite	87 (1.22)
Calcium compounds	24 (1.31)	Titanium compounds	86 (1.20)
Silver compounds	22 (1.20)	Curcumin	65 (0.91)
Silica compounds	21 (1.15)	Silica compounds	62 (0.87)
Cloisite	17 (0.93)	Beeswax	61 (0.85)
Graphene	14 (0.76)	Glutaraldehyde	52 (0.73)
Nisin	9 (0.49)	Graphene	45 (0.63)
Carbon nanotubes	9 (0.49)	Carvacrol	42 (0.59)
Beeswax	9 (0.49)	Guar gum	39 (0.55)
Other	597 (32.57)	Other	2421 (33.92)
Not available^a^	8	Not available	57

a “Not available” represents the number of articles for which information could not be obtained.

Glycerol corresponded to 29.1% and 26.9% of the mention of additives between 2013 and 2018 and between 2019 and 2024, respectively. This prevalence can be attributed to the plasticizing properties of this additive, which increase the flexibility and reduce the fragility of biopolymers such as starch and chitosan. In addition, glycerol is biobased, widely available, inexpensive, and efficient. Sorbitol, polyethylene glycol, and Tween are other plasticizers employed. Glycerol was used in 83% of the studies reviewed by Sampaio et al. (2023), and other identified plasticizers included polyols such as sorbitol, ethylene glycol, propylene glycol, and polyethylene glycol, which are also easily incorporated into different matrices.^65^

Given that glycerol has been the most used plasticizer to prepare sustainable packages and utensils, we analyzed its concentration as described in the articles. The articles mentioning glycerol and published between 2013 and 2018 (*n* = 339) reported average and mean glycerol concentrations of 32.77% and 30%, respectively (where % refers to *g*_glycerol_/100 *g*_polymer_ in the matrix). In turn, the articles mentioning glycerol and published between 2019 and 2024 (*n* = 1208) reported average and mean glycerol concentrations of 31.81% and 30%, respectively. The constant median glycerol concentration of 30% in both time intervals suggests that, despite variations, studies maintained this additive within a standard concentration range, which highlights that it is stable in sustainable materials. However, the ideal glycerol concentration may vary depending on the formulation composition and the final material application. For example, a central rotatable composite design indicated that 36.0% glycerol is ideal for films consisting of myofibrillar proteins derived from fish byproducts, resulting in improved mechanical properties and water resistance.^87^ On the other hand, univariate experiments determined that 30.0% glycerol is optimal for chicken skin gelatin-tapioca starch films and plasticized alginate films.^88,89^ It should be noted, however, that these values are only initial recommendations as each material type requires a specific amount of plasticizer. For instance, for carboxymethyl cellulose edible coatings incorporating ZnO nanoparticles, 75% glycerol was used, whereas for chitosan-cassava starch films with TiO_2_, only 20% glycerol was applied.^90,91^ The desired properties for the obtained materials will also define them—the obtained materials’ stiffness, strength, deformation, water absorption, and swelling, for instance.

Vegetable oils, essential oils, and natural extracts appear as the second most employed additives. These compounds are valued for their antimicrobial and antioxidant properties, which potentially extend the shelf life of packaged products. The percentage of mentions of these additives increased from 12.0% between 2013 and 2018 to 14.7% between 2019 and 2024, reflecting growing interest.

Meanwhile, lignocellulosic fibers and/or particles accounted for 2.4% and 4.2% of the mention of additives between 2013 and 2018 and between 2019 and 2024, respectively. This indicates that interest in natural reinforcing materials has increased and that biomass has become increasingly valorized. Moreover, by adding biomass as filler, the quantity of applied biopolymer is reduced, which is relevant when considering challenges such as cost and potential competition of resources among applications (e.g., land use for food or materials). Biobased refers to materials derived from biological sources, while renewable refers to resources that can naturally replenish within a human time scale. Lignocellulosic fibers are biobased, biodegradable, and renewable, and may improve the mechanical properties of the sustainable materials to which they are added.^12,75^

Incorporating metallic compounds such as silver, zinc, and titanium into sustainable materials is an interesting approach because they exhibit antimicrobial and structural reinforcement properties.^92,93^ Using clays, particularly montmorillonite (especially in the form of nano- or microparticles), is a relevant strategy in the case of sustainable composites. Montmorillonite is low-cost, displays mechanical reinforcement properties, which make sustainable materials more rigid and resistant, and enhances gas and moisture barrier properties. Its large surface area and ability to interact with polymer matrices also favor their uniform dispersion, contributing to the improved performance. These characteristics make montmorillonite a promising choice for formulating nanocomposites for sustainable materials applications. Clays such as cloisite, halloysite, zeolite, bentonite, and kaolin can also be added to sustainable packaging and utensils.^94–96^ However, the addition of both metallic compounds and clays can jeopardize the sustainability of the obtained materials, with some points, such as their effect on the end-of-life, still open. Moreover, migration studies are needed to guarantee safer applications when they are in contact with foods.

Citric acid stands out as a multifunctional additive for developing sustainable materials. Its antimicrobial potential helps to preserve food, while its ability to act as a plasticizer makes packaging flexible and malleable. Moreover, citric acid can be used as an effective and low-cost cross-linker.^21^

Glutaraldehyde is one of the most frequently reported cross-linkers due to its ability to form covalent bonds between molecules in polymer matrices, thereby enhancing the mechanical strength and thermal stability of materials. Its effectiveness as a cross-linker has made it a common choice in academic studies aimed at improving structural stability and durability. However, its well-documented toxicity raises serious concerns about its suitability, due to potential migration risks.^47,97^ This limitation likely explains the notable decrease in its reported use, from 1.3% of mentions between 2013–2018 to 0.7% between 2019–2024. The reliance on glutaraldehyde exemplifies a critical trade-off in the field: achieving superior functional properties versus maintaining nontoxicity and true sustainability. Its reported decline underscores the urgent need for safer biobased alternatives. For instance, citric acid, tannic acid, or genipin can be alternatives, which are also documented in this review. When glutaraldehyde is applied, extreme caution and extensive migration testing are required, further reinforcing its limited potential for real-world sustainable applications.

The significant proportion of additives classified as ‘Other’ (32.6% from 2013 to 2018 and 33.9% from 2019 to 2024) underscores a trend of exploration and innovation in the sustainable materials field. This category encompasses emerging compounds, often tailored for niche applications and not yet widely adopted. Although the ‘Other’ group represents a substantial share, most individual additives within it accounted for less than 0.5% of the total mentions, indicating only marginal representation. These included a diverse range of substances such as inorganic compounds and organometallic compounds such as Al_2_(SO_4_)_3_, magnesium carbonate, Fe_2_O_3_, kaolin, laponite, kimberlite, halloysite, zeolite, talc, borax, lithium chloride, Au^+^, selenium nanoparticles, cobalt­(II) stearate, and magnesium stearate; organic acids such as gallic acid, tannic acid, lactic acid, maleic acid, humic acid, ferulic acid, cinnamic acid, caproic acid, and ascorbic acid; plasticizers, cross-linkers, and stabilizers including bis­(2-ethylhexyl) adipate, bis­(2-ethylhexyl) sebacate, glucose, fructose, glyoxal, epichlorohydrin, xilitol, and Irganox; bioactive, antimicrobial, and antioxidant agents such as cinnamaldehyde, eugenol, α-tocopherol, vanillin, quercetin, propolis byproducts, caffeine, benzoate, epigallocatechin gallate, bixin, and genipin; proteins, enzymes, and biopolymers including ε-polylysine, transglutaminase, lactoferrin, and lipase.

### 3.6 Techniques to Characterize Sustainable and Single-Use Utensils

Table 4 lists the main techniques used to characterize sustainable materials developed between 2013 and 2018 and between 2019 and 2024. Characterization techniques are essential for evaluating the physical, chemical, structural, and functional properties.

**Table 4:** Techniques to Characterize Sustainable Packaging and Single-Use Utensils: Comparison of Articles Published between 2013 and 2018 and between 2019 and 2024

2013–2018		2019–2024	
Total	%	Total	%
Tensile properties	922 (11.60)	Tensile properties	3346 (9.38)
SEM	606 (7.62)	FTIR	2834 (7.94)
FTIR	540 (6.79)	SEM	2626 (7.36)
Water vapor transmission rate	526 (6.62)	Water vapor transmission rate	2189 (6.14)
Thickness	457 (5.75)	TGA	1916 (5.37)
TGA	428 (5.38)	Thickness	1684 (4.72)
XRD	402 (5.06)	XRD	1573 (4.41)
UV–vis properties, opacity, transmittance	344 (4.33)	UV–vis properties, opacity, transmittance	1503 (4.21)
Solubility in water	332 (4.18)	Solubility in water	1288 (3.61)
DSC	291 (3.66)	Antimicrobial activity	1269 (3.56)
Color	260 (3.27)	Contact angle with liquid water	1271 (3.56)
Moisture content	212 (2.67)	DSC	1208 (3.39)
Antimicrobial activity	208 (2.62)	Color	1097 (3.07)
Contact angle with liquid water	181 (2.28)	Biodegradability or disintegration	1007 (2.82)
Biodegradability or disintegration	177 (2.23)	Moisture	1006 (2.82)
Oxygen permeability	174 (2.19)	Performance tests	956 (2.68)
Water absorption	137 (1.72)	Swelling ability	827 (2.32)
Performance tests	136 (1.71)	DPHH test	809 (2.27)
Dynamic mechanical and DMTA	105 (1.32)	Oxygen permeability	694 (1.95)
TEM	101 (1.27)	FESEM	383 (1.07)
FESEM	94 (1.18)	AFM	325 (0.91)
AFM	94 (1.18)	Release and migration tests	315 (0.88)
Density	87 (1.09)	ABTS test	296 (0.83)
Swelling ability	82 (1.03)	Density	285 (0.80)
DPPH test	77 (0.97)	TEM	261 (0.73)
Release and migration tests	66 (0.83)	Dynamic mechanical and DMTA	250 (0.70)
Water vapor uptake	62 (0.78)	NMR	244 (0.68)
NMR	62 (0.78)	Rheological properties	154 (0.43)
Other	788 (9.91)	Other	4062 (11.39)
Not available^a^	17	Not available	46
Total	7951	Total	35678
Average	6.4	Average	7.9
Median	6	Median	8
Range	1–17	Range	1–22

a “Not available” represents the number of articles for which information could not be obtained.

Between 2013 and 2018, each article reported an average of 6.4 and a median of 6 characterization techniques. Between 1 and 17 characterization techniques were employed per article. This could be related to an earlier stage of consolidation of characterization techniques, when greater emphasis was placed on fundamental properties such as mechanical strength, thermal stability, and moisture barrier, which are necessary to validate the use of sustainable materials under practical conditions. Between 2019 and 2024, the values changed to an average of 7.9 and a median of 8 characterization techniques, with an expanded range of 1 to 22 techniques. This indicates that more complex, specific, and established characterization techniques were incorporated in the studies as a natural evolution of scientific development.

Techniques to determine the tensile properties were the most used between 2013 and 2018 (11.6% of the mentions of characterization techniques in this time interval) and between 2019 and 2024 (9.4% of the mentions of characterization techniques in this time interval). These techniques are fundamental for evaluating mechanical strength and elasticity, which are essential for ensuring durability and functionality. The widespread use of these techniques is directly linked to the fact that polymer films were the most investigated sustainable materials between 2013 and 2024. Films require appropriate strength and flexibility to withstand handling, transportation, and storage processes, and tensile testing is a reliable indicator of these properties. Between 2019 and 2024, the use of these techniques decreased slightly, which reflects that the characterization techniques became diversified, and that complementary techniques addressing other aspects emerged.^98,99^ Even so, we highlight the need for more detailed knowledge in relation to the mechanical properties of sustainable materials, including other mechanical assays further than tensile tests (bending, flexion, torsion, puncture, trouser tear, compression) and in different conditions (temperature, equilibrium moisture, aging), therefore supporting real applications.

Fourier-transform infrared spectroscopy (FTIR) continued being relevant between 2019 and 2024 (7.9% of the mentions of characterization techniques in this time interval), becoming the second most employed characterization technique because it is simple and low-cost and identifies functional groups and chemical interactions in materials. FTIR generates a characteristic spectrum in the infrared region, which acts as a “chemical fingerprint” of the material. This allows not only components to be qualitatively identified but also structural changes induced by modification or addition processes to be evaluated. Additionally, FTIR is versatile, which makes it particularly useful for investigating polymers and composites used in sustainable materials. However, complementary characterization techniques, such as Raman spectroscopy, are rarely applied; therefore, further developments are needed to understand how molecular and material structures are related to material properties.

Scanning Electron Microscopy (SEM) was extensively used between 2013 and 2018 and between 2019 and 2024 (7.6% and 7.4% of the mentions of characterization techniques in these time intervals, respectively), being a consolidated technique for analyzing microstructure and morphology. Along with Field Emission Scanning Electron Microscopy (FESEM), Transmission Electron Microscopy (TEM), and Atomic Force Microscopy (AFM), these techniques provide detailed images of the material’s surface, being used to evaluate characteristics such as pore distribution, homogeneity, and potential structural defects.^100^ These techniques also help micro- and nanoscale features to be correlated with the macroscopic results of other techniques, including mechanical strength, total porosity, and water absorption tests, facilitating the interpretation and optimization of properties. However, it is important to highlight some limitations such as the very narrow field and the limitation of surface observation.

Ultraviolet–Visible Spectroscopy (UV–vis), along with opacity and transmittance analyses, helps to evaluate optical properties, while consumer aesthetic preferences are met. Packaging appearance affects consumer perception and directly influences the purchasing intent, especially in the food and cosmetic sectors.^6,101^

Characterization techniques such as Thermogravimetric Analysis (TGA) and Differential Scanning Calorimetry (DSC) help to determine thermal stability and phase transitions. TGA assesses mass loss as a function of temperature, identifying the thermal degradation and composition of the materials. DSC provides information on structural (crystallinity) and endothermic and exothermic processes, such as fusion, crystallization, and glass transition, which helps to understand the thermal behavior of polymers used in sustainable materials. Thermal stability ensures that a sustainable package maintains its mechanical and functional properties during processing, storage, and transportation. Absence of thermal degradation is vital for preserving packaged product quality and avoiding quality and safety issues. In addition, thermally resistant, sustainable materials are more versatile and suitable for applications involving extreme temperature variations, such as refrigerated or heated preparation or storage.^102,103^

Water and moisture resistance is also essential and a challenge for materials based on biopolymers, which justifies the range of techniques that test mainly the Contact Angle, Swelling, Solubility, and Water Vapor Transmission Rate (WVTR). These tests are used to evaluate the material’s affinity with water (liquid or vapor, surface or bulk), reflecting progress in efforts to enhance protection against moisture.^104^ Among these, the WVTR test was the most frequently applied characterization technique between 2013 and 2018 (6.6% of all mentions of characterization techniques in this period) and between 2019 and 2024 (6.1% of mentions). Contact angle with water also stood out in these intervals (2.3% and 3.6% of mentions of characterization techniques, respectively). Determining the solubility in water complements the analysis because it indicates whether sustainable packaging is chemically and physically stable in aqueous environments. Excessive solubility is undesirable, especially for sustainable packaging that is in contact with moist products or exposed to variable storage conditions.

Analyzing the antioxidant activity through DPPH (1,1-Diphenyl-2-picrylhydrazyl radical scavenging test), or ABTS (2,2′-azino-bis­(3-ethylbenzothiazoline-6-sulfonic acid) radical scavenging test is important in the case of bioactive sustainable packaging.^105^ Meanwhile, characterization techniques such as Nuclear Magnetic Resonance (NMR) and X-ray Diffraction (XRD) appear less frequently but remain relevant for more detailed structural analysis.

Regarding barrier properties, a significant gap is observed in the evaluation of gas permeability in sustainable packaging materials. Oxygen permeability represented only 2.2% of the mentions of characterization techniques between 2013–2018 and 1.9% between 2019–2024, indicating limited attention to this critical property for food preservation. Air, CO_2_, and NH_3_ permeability were reported even less frequently and were grouped under the “Other” category. This lack of investigation into the barrier properties against a wider range of gases represents a major limitation for the practical application of these materials, as such characteristics are determinants for the functional performance of packaging in real-world storage, distribution, and product quality monitoring conditions.

Despite their critical importance for safety assessment, release and migration tests remain significantly understudied. These tests accounted for a mere 0.8% and 0.9% of all characterization techniques mentioned in the 2013–2018 and 2019–2024 periods, respectively. The interaction of the obtained materials with food and the environment, through release and migration tests, is used to evaluate whether compounds are transferred from materials to the external environment or packaged product. These tests help to ensure that food is safe and that the functional properties are controlled. They help to identify the potential release of toxic or contaminating components that could jeopardize product safety. Besides that, these tests assist in evaluating the effectiveness of additives incorporated into sustainable materials, such as antimicrobials, antioxidants, or other bioactive compounds.^98,106^

The following sections will detail the techniques for evaluating sustainable packaging antimicrobial activity, sustainable packaging applications, and biodegradability or disintegration. The various techniques available for characterizing sustainable packaging reflect continuous evolution and innovation in sustainable packaging research, which increasingly focuses on meeting specific functional requirements and addressing environmental concerns. Integrating advanced materials and characterization techniques demonstrates a commitment to developing more efficient, reliable, and eco-friendly packaging solutions that align with global sustainability goals.

### 3.7 Sustainable Packaging Antimicrobial Activity

Table 5 provides an overview of the microorganisms used to evaluate the sustainable packaging antimicrobial activity between 2013 and 2018 and between 2019 and 2024. Between 2013 and 2018 and between 2019 and 2024, 1022 (83.1%) and 3,190 (70.1%) of the articles did not report any antimicrobial activity tests, respectively.

**Table 5:** Microorganisms Used to Evaluate Sustainable Packaging Antimicrobial Activity: Comparison of Articles Published between 2013 and 2018 and between 2019 and 2024^a^

2013–2018		2019–2024	
Microorganism	Total (%)	Microorganism	Total (%)
*Escherichia coli* (−)	148 (22.91)	*Escherichia coli* (−)	1033 (30.28)
*Staphylococcus aureus* (+)	126 (19.50)	*Staphylococcus aureus* (+)	918 (26.91)
*Listeria monocytogenes* (+)	56 (8.67)	*Salmonella enterica* (−)	197 (5.77)
*Salmonella enterica* (−)	56 (8.67)	*Listeria monocytogenes* (+)	172 (5.04)
*Bacillus cereus* (+)	32 (4.95)	*Pseudomonas aeruginosa* (−)	165 (4.84)
*Pseudomonas aeruginosa* (−)	21 (3.25)	*Bacillus subtilis* (+)	101 (2.96)
*Bacillus subtilis* (+)	21 (3.25)	*Candida albicans* (F)	95 (2.78)
*Aspergillus niger* (F)	16 (2.48)	*Bacillus cereus* (+)	84 (2.46)
*Candida albicans* (F)	8 (1.24)	*Aspergillus niger* (F)	56 (1.64)
*Listeria innocua* (+)	7 (1.08)	*Penicillium spp.* (F)	45 (1.32)
*Pseudomonas fluorescens* (−)	5 (0.77)	*Enterococcus faecalis* (+)	33 (0.97)
*Shewanella putrefaciens* (−)	5 (0.77)	*Listeria innocua* (+)	29 (0.85)
*Vibrio parahemolyticus* (−)	5 (0.77)	*Klebsiella pneumoniae* (−)	27 (0.79)
*Clostridium perfringens* (+)	5 (0.77)	*Pseudomonas fluorescens* (−)	16 (0.47)
*Penicillium expansum* (F)	4 (0.62)	*Aspergillus flavus* (F)	15 (0.44)
*Streptococcus mutans* (+)	3 (0.46)	*Staphylococcus epidermidis* (+)	14 (0.41)
*Enterobacter aerogenes* (−)	3 (0.46)	*Micrococcus luteus* (+)	14 (0.41)
*Proteus mirabilis* (−)	3 (0.46)	*Saccharomyces cerevisiae* (F)	13 (0.38)
Other	122 (18.89)	Other	385 (11.28)
Not available	13	Not available	42

a (+) Gram-positive bacteria, (−) Gram-negative bacteria, (F) Fungus. “Not available” represents the number of articles for which information could not be obtained.

Over time, *Escherichia coli* and *Staphylococcus aureus* were increasingly investigated and remained the most tested. Together, they accounted for 42.41% and 57.19% of the mentions of antimicrobial tests between 2013 and 2018 and between 2019 and 2024, respectively, which underscores that they are relevant testing models. The fact that studies investigating *E. coli* prevailed reflects that this species is a microbiological indicator of food safety, while *S. aureus* is studied because it forms biofilms and contaminates perishable foods.^107,108^ Microorganisms, such as *Salmonella enterica* and *Listeria monocytogenes*, have gained prominence, given that they are important foodborne pathogens.^109^

Other microorganisms, including *Penicillium spp.*, *Klebsiella pneumoniae*, and *Micrococcus luteus*, appeared more frequently in articles published between 2019 and 2024, which reflects that the scope of research was expanded to include species relevant to specific environments, packaging types, or applications.

The numerous articles exploring antimicrobial activity in sustainable packaging are likely linked to the prevalence of sustainable packaging with active properties, such as controlled release of antimicrobial or antioxidant agents. These properties are useful in the food industry, which prioritizes extended product shelf life and microbiological safety. Incorporating bioactive compounds, like natural extracts, essential oils, or synthetic compounds, into sustainable packaging has been known to protect products against spoilage and pathogenic microorganisms.^44,110,111^

### 3.8 Tests to Assess the Sustainable Packaging and Single-Use Utensils’ Biodegradability or Disintegration

Assessing sustainable packaging biodegradability ensures that the packaging decomposes safely and efficiently in natural environments, in a reasonable timeline, especially if it is labeled as biodegradable. Biodegradability tests verify whether sustainable packaging decomposes appropriately under specific conditions (e.g., in soil, water, or controlled environments). Biodegradable packaging is advantageous over conventional plastics, which have been persistent in the environment for centuries, thereby contributing to the accumulation of plastics and microplastics in oceans. Interest in biodegradable packaging has increased over recent years, reflecting heightened awareness of the environmental pollution caused by conventional plastics, particularly single-use plastics. However, biodegradation tests are complex and time-consuming, which can explain why they are not often conducted in the evaluation of scientific articles. Table 6 presents the main tests used to assess the biodegradability or disintegration. Between 2013 and 2018 and between 2019 and 2024, 1,052 (85.6%) and 3,453 (77.4%) studies did not assess biodegradability or disintegration, respectively.

**Table 6:** Tests to Assess Sustainable Packaging and Single-Use Utensils’ Biodegradability or Disintegration: Comparison of Articles Published between 2013 and 2018 and between 2019 and 2024

2013–2018		2019–2024	
Biodegradability or disintegration test	Total (%)	Biodegradability or disintegration test	Total (%)
Simulated soil burial	103 (55.68)	Simulated soil burial	717 (67.83)
Enzymatic degradation	15 (8.11)	Soil burial under natural conditions	52 (4.92)
Microbial degradation	11 (5.95)	ISO 20200 (composting conditions)	37 (3.50)
ISO 20200 (composting conditions)	9 (4.86)	Microbial degradation	34 (3.22)
Soil burial under Natural conditions	8 (4.32)	Solution, hydrolytic degradation	34 (3.22)
Solution, hydrolytic degradation	8 (4.32)	Enzymatic degradation	27 (2.55)
ISO 14855 (aerobic biodegradation)	7 (3.78)	ASTM D 5988 ((aerobic biodegradation)	25 (2.37)
ASTM G160–03 (microbiological susceptibility)	2 (1.08)	ISO 14855 (aerobic biodegradation)	18 (1.70)
ASTM D 5988 (aerobic biodegradation)	2 (1.08)	Sea water, lake, river	17 (1.61)
		ASTM D 53338 (aerobic biodegradation)	9 (0.85)
Other	20 (10.81)	Other	87 (8.23)
Not available^a^	14	Not available	41

a “Not available” represents the number of articles for which information could not be obtained.

Standardized tests are crucial for classifying materials on the basis of their degradation properties, facilitating certifications, and the development of regulations, such as ISO (International Organization for Standardization) 20200, ISO 14855, ASTM (American Society for Testing and Materials) D 5988, and ASTM D 53338. These standards provide consistent evaluations to ensure that sustainable packaging is suitable for the intended end-of-life scenarios, such as industrial composting and marine degradation. For example, ISO 20200, the most mentioned standardized test, measures the rate at which plastics degrade in simulated soil under laboratory conditions during a minimum test period of 45 days.^112,113^ On the other hand, the standard evaluation only allows standard conclusions, which do not necessarily consider the variety of possible scenarios of the materials endline—including the countless number of soil and microbiome, water systems (composition, salinity, microbiology, movement), temperature and weather, among others, around the world.

Simulated soil burial tests were the most used in both time intervals and increased from 55.7% of the mention of biodegradability or disintegration tests between 2013 and 2018 to 67,8% between 2019 and 2024. Simulated soil burial involves controlled tests where sustainable packaging is buried in pots or containers with defined degradation parameters, such as temperature, humidity, and pH. While the tests simulate natural conditions, they are not tied to specific standards and serve to test biodegradability or disintegration under controlled experimental settings.^114–116^ Simulated soil burial is interesting for comparing treatments to control, but comparison with literature findings might be difficult. Conversely, “Soil burial under natural conditions” involves tests conducted directly in natural environments without stringent condition controls. Sustainable packaging is buried in soil and exposed to natural factors, such as climatic variations, microorganism activity, and other environmental elements. This approach offers a more realistic view of how sustainable packaging behaves when it is discarded in various ecosystems without experimental interference.^56,117^ However, the results depend on the selected microsystem. Therefore, although treatments can be compared to the control, a comparison with the literature can be difficult or limited to specific systems.

More specific tests, such as ASTM D 5988, which gauges aerobic biodegradation by measuring carbon dioxide release over time when sustainable packaging interacts with soil, have become relevant, corresponding to 1.1% and 2.4% of the mentions between 2013 and 2018 and between 2019 and 2024, respectively. Moreover, tests were diversified and include enzymatic degradation, which employs enzymes to simulate natural sustainable packaging decomposition; marine, lake, and river degradation tests, which assess how plastics break down in aquatic environments; and microbial degradation, which uses specific microorganisms to evaluate packaging biodegradability or disintegration. Additionally, tests such as solution degradation and hydrolytic degradation assess how sustainable packaging reacts in the presence of water or chemical solutions to accelerate decomposition. These tests are vital for understanding how biodegradable packaging impacts the environment. The growing application of innovative biodegradability or disintegration tests reflects technological and scientific advancements in tailoring them to meet the contemporary need to assess sustainable packaging.^53,113,118,119^

### 3.9 Strategies to Improve Sustainable Materials Properties

Table 7 presents the key strategies to improve sustainable material properties mentioned between 2013 and 2018 and between 2019 and 2024. Between 2013 and 2018, and between 2019 and 2024, 151 (12.15%) and 361 (8.0%) articles did not report a specific strategy, respectively. Other specifications highlighted by the authors are presented (use of residue, industrial byproducts, nanostructured polymers, and nanostructured additives), with the percentages calculated based on the total number of articles mentioning them relative to the total number of mentions of any strategy to improve materials properties in the analyzed time interval.

**Table 7:** Strategies to Improve Sustainable Materials Properties: Comparison of Articles Published between 2013 and 2018 and between 2019 and 2024

	2013–2018	2019–2024
Strategy	Total (%)	Total (%)
Polymeric blend	490 (34.78)	1752 (31.51)
Functional (active, bioactive, biotic, edible, intelligent, pro-oxidant, antioxidant)	332 (23.56)	1601 (28.79)
Other composites, filler	305 (21.65)	1199 (21.56)
Polymer modification	177 (12.56)	577 (10.38)
Multilayer	57 (4.05)	244 (4.39)
Multivariate experimental design	47 (3.34)	172 (3.09)
Reuse	1 (0.07)	16 (0.29)
Not Available^a^	1	18
**Other specifications**		
Residue, industrial byproduct	88 (7.08)^b^	490 (10.92)^b^
Nanostructured polymers	111 (8.89)^b^	524 (11.68)^b^
Nanostructured additives	115 (9.26)^b^	884 (19.70)^b^

a “Not available” represents the number of articles for which information could not be obtained.

b Percentage calculated based on the total articles from the period.

Polymeric blends were the most studied strategy to improve materials properties in both time intervals, but the percentage of mentions decreased from 34.8% between 2013 and 2018 to 31.5% between 2019 and 2024. This may be related to diversification. The predominance of polymeric blends reflects that this strategy is versatile for enhancing sustainable materials’ mechanical, chemical, and physical properties, which are essential for functionality. Moreover, similarly to the previous discussion about additive and biobased fillers, blending can reduce the applied quantity of a given biopolymer, which can be relevant when considering challenges such as cost and potential competition of resources among applications.

Regarding functional materials, which include active, bioactive, biotic, edible, intelligent, pro-oxidant, and antioxidant classifications, the percentage of mentions increased from 23.6% between 2013 and 2018 to 28.8% between 2019 and 2024. This reflects the growing demand for sustainable materials that perform additional functions, such as extending product shelf life, monitoring storage conditions, providing bioactive or biotic components, and delivering other direct benefits to consumers.^120–122^

Notably, the percentage of mentions of the use of residues and industrial byproducts to improve sustainable materials properties, often to replace part of pure polymers, rose from 7.0% between 2013 and 2018 to 10.9% between 2019 and 2024. This indicates progress in developing solutions aligned with the principles of the circular economy given that discarded materials are used as raw materials for new applications. Simultaneously, the percentage of mentions of composite and filler materials to improve sustainable materials properties remained stable at 21.6% in both periods (2013–2018 and 2019–2024), indicating interest in exploring various components to reinforce materials properties.

Multivariate experimental design experiments are a powerful tool to optimize and improve the development of new materials, but they are underused and under-reported in the literature. These experiments allow multiple variables and their interactions to be analyzed simultaneously, providing a comprehensive understanding of the factors that affect the product performance and efficiency.^68,123^ For instance, in studies using polymeric blends or functional materials, multivariate design helps to identify the best proportions among components so that optimal mechanical properties, such as tensile strength or flexibility, are achieved, while biodegradability or recyclability is maintained. However, this type of experiment has been hardly employed (approximately 3%), probably because a multifactor evaluation requires a large number of treatments and a large amount of experimental work, which certainly challenges researchers.

Table 7 shows that the use of nanostructured polymers and nanostructured additives to improve sustainable materials properties increased between the analyzed time intervals. Nanostructured polymers represented 8.9% and 11.7% of the mentions of strategies between 2013 and 2018 and between 2019 and 2024, respectively, which indicates moderate growth. On the other hand, the use of nanostructured additives increased substantially, from 9.3% between 2013 and 2018 to 19.7% between 2019 and 2024. These results reflect that nanomaterials are being increasingly incorporated into sustainable materials to improve their mechanical, barrier, and functional properties. Nanostructured polymers are widely applied to reduce gas and moisture permeability and to enhance the strength and durability of materials. Nanostructured additives, such as metallic nanoparticles and clays, stand out for their antimicrobial properties, structural reinforcement, and potential to modify the thermal and optical characteristics of materials. The more pronounced increase in the use of nanostructured additives suggests a greater focus on specific applications, including enhanced protection against microbiological contamination and improved functional barriers.^124–126^ This expanded use also reflects that manufacturing technologies have advanced and the cost of some nanomaterials has decreased in recent years, which has made them more accessible and viable for commercial applications. However, similarly to discussed before, migration studies are needed to guarantee safer applications when in contact with foods, and the impact of those approaches on the end-of-life of materials is needed to claim sustainability.

### 3.10 Sustainable Packaging Performance during Application

Figure 4 shows sustainable packaging applications across different products published between 2013 and 2018 and between 2019 and 2024. The applications described in this section are primarily proof-of-concept studies. While they demonstrate the potential of the developed materials for food packaging, most of these systems are at a low Technology Readiness Level (TRL) and are not yet commercially available. Therefore, the reported results should be interpreted as indicative of future possibilities rather than current market-ready solutions.

**Figure 4:**
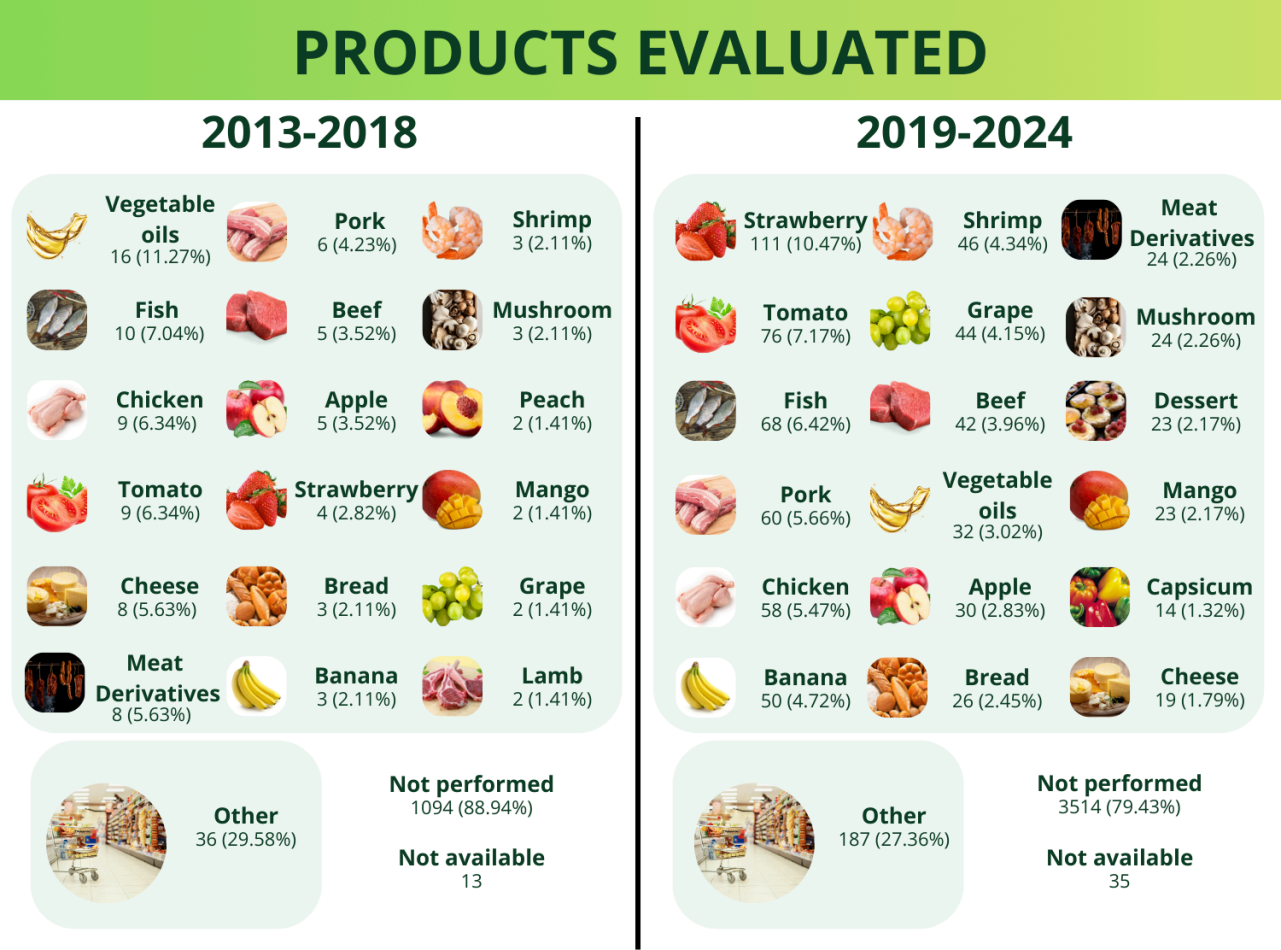
Sustainable packaging applications: comparison of articles published between 2013 and 2018 and between 2019 and 2024.

Between 2013 and 2018 and between 2019 and 2024, 10094 (88.9%) and 3,514 (79.4%) of the articles did not report applications. On going from the first to the second time interval, mention of applications increased by 9.5%. Products such as strawberries, fish, chicken, and tomatoes were among the most common choices for testing, with strawberries standing out between 2019 and 2024 (10.5% of the mentions of applications). Meanwhile, between 2013 and 2018, vegetable oils, fish, chicken, and tomatoes (11.3%, 7.0%, 6.3%, and 6.3% of the mentioned applications, respectively) were primarily tested.

Most articles focused on the food sector, particularly perishable items. These products are highly susceptible to rapid degradation, which is both a challenge for packaging performance and a source of waste (in contrast to long-term applications for the materials, food packages are quickly disposable). These characteristics make them ideal for studying the effectiveness of sustainable packaging. During their shelf life, key food quality parameters—such as freshness, moisture content, microbial growth, and nutritional value—can be closely monitored. This allows researchers to directly compare the performance of sustainable packaging with that of conventional plastics, evaluating factors such as preservation efficiency and barrier properties.

According to estimates by the Food and Agriculture Organization of the United Nations and the United Nations Environment Programme, approximately 19% is wasted along the food supply chain.^127^ This highlights significant inefficiencies in handling, storage, and transportation, underscoring the need for improved practices and technologies to reduce waste in this critical sector.^128^ Extending food shelf life helps to reduce not only waste but also the frequency of food disposal due to quality or expiration issues. This benefits the supply chain, reduces pressure on agricultural production, and minimizes the environmental impact.^129,130^

In the literature, we can also find tests conducted on nonfood products, including items intended for e-commerce, medical, pesticide, and hygiene product packaging, grouped with other foods under the category “Others”. When considering the entire period from 2013 to 2024, only six articles were identified that specifically focused on testing nonfood products. This limited number highlights a potential gap in research addressing the application of these materials in nonfood contexts. Despite this gap, the existing studies broaden the application scope of sustainable packaging, demonstrating its versatility and potential to serve various market sectors beyond the food industry.^131,132^

### 3.11 Challenges in Developing Sustainable Packaging

On the basis of the points discussed above, challenges remain to be overcome for sustainable packaging to be broadly accepted in the market. Economic feasibility is a critical component of sustainability, placing sustainable materials at a competitive disadvantage compared to that of traditional polymers. For eco-friendly materials to become economically viable and accessible to consumers of all classes, their price must approach the price of conventional materials without affecting sustainability. While many of the discussed solutions show environmental advantages (e.g., biodegradability, biobased feedstocks, lower toxicity), their current high production cost limits large-scale application. Thus, these materials are potentially sustainable in terms of environmental performance, but their sustainability will only be achieved when cost-effective production and scalability are addressed.^133,134^

The sustainable materials performance, when compared to traditional materials, is another point to be improved. While mechanical behavior is a significant barrier to entry in the market, it is not the only functional limitation. For widespread adoption, the performance of sustainable packaging must also overcome challenges related to gas barrier properties, thermal stability, and susceptibility to moisture, which are currently often inferior to those of conventional polymers. These shortcomings can compromise food preservation, limit shelf life, and restrict applications that require high resistance to environmental conditions.

Another critical challenge is the competition for land use, which may be allocated either for producing food, raw materials for materials production, or any other application (biofuel, forest, etc.). This dilemma demands balanced solutions that prioritize food security, without hindering innovation in sustainable materials. In addition, the variability in raw material sources and processing methods may further affect the consistency of these properties. Therefore, addressing these limitations through innovative formulations, nanostructured additives, or hybrid systems is essential to ensure that sustainable packaging can match the performance of traditional plastics while maintaining its environmental advantages.

The fact that research on films accounted for over 90% of the articles highlights a gap in the development of solutions for other disposable items including rigid packages, bottles, straws, cutlery, and trays. While films are widely used and considerably impact plastic pollution, it must be recognized that other disposable items also contribute to environmental issues as well as the real-life need for different materials for different applications. Straws, cutlery, and trays, for instance, are often improperly discarded, especially in environments such as beaches, parks, and events, where managing waste is more problematic.

Although the casting method is currently the most employed to prepare sustainable materials, mainly because films are the most studied type, research into other methods with potential industrial application must be expanded, particularly extrusion and thermocompression. Diversifying production methods can contribute to reducing costs and increasing efficiency in manufacturing, also allowing for obtaining the variety of products applied in different contexts.

Developing functional materials by incorporating innovative features such as antimicrobial, antioxidant, or sensory properties can serve as a decisive competitive differentiator, attracting and retaining consumer attention.

Finally, environmental footprint, economic, and social assessment are needed for increasing TRLs toward a broad application of sustainable packages and utensils, considering the complete system (for example including the discussions about additives) and techniques such as multiple biodegradation scenarios, Life-Cycle Assessment (LCA), Life Cycle Costing (LCC), Techno-Economic Analysis (TEA) and the expectations and behaviors of all the involved stakeholders (consumers, producers, police makers, etc.).

Facing these challenges requires a concerted effort among researchers, industry, and public policies to make sustainable packages and utensils not only a viable alternative but also the preferred choice in the global market.

### 3.12 Limitations of the Study

Our search strategy may have overlooked certain evidence. In the selection process, we opted to include only studies that addressed specific terms, which may limit the breadth of our findings. The application of exclusion criteria during selection may contribute to the vulnerabilities in our review. Regarding the specification of terms, this methodology facilitated a more detailed understanding within this field of study.

The use of a single database (Scopus) may have excluded relevant studies indexed only in other databases such as Web of Science, PubMed, or Google Scholar. Even so, Scopus was one of the most relevant scientific databases in the study period, which justifies our approach.

Furthermore, the review exclusively considered peer-reviewed articles, excluding other valuable sources of information such as patents, technical reports, industry white papers, and conference proceedings. This is clearly stated in the text and demonstrates scientific development rather than innovation or market aspects.

Additionally, the study included only articles published in English, Portuguese, and Spanish, which may have excluded important contributions in other languages. Even so, the sum of the scientific papers published in these three languages can be considered to be internationally relevant.

## 4 Conclusion

Analyzing the data on preparation methods, raw materials, and additives used to prepare sustainable packages and utensils and applications has revealed that research into this field has increased and advanced over the years. Between 2013 and 2018, polymers such as starch and cellulose as well as additives like glycerol, which provide essential flexibility and plasticizing properties, were primarily investigated. Between 2019 and 2024, nanomaterials, such as nanostructured polymers and nanostructured additives, were increasingly employed, which reflects growing interest in technological innovations that enhance sustainable materials functionality, particularly in terms of resistance and barriers against external agents. Between 2013 and 2024, film was the most developed sustainable material type, which shows that more efforts are needed to produce other types of materials, such as rigid materials, bottles, foam trays and cups, straws, and bags. More applications of fully developed packages are necessary to provide new products for the industry. Despite the growing use of additives and new materials, water resistance remains the biggest concern for sustainable materials. This justifies the wide range of tests employed, including tests on water absorption and the water vapor transmission rate. Such resistance is essential for maintaining the integrity of materials under storage and transport conditions. Additionally, biodegradability or disintegration, antimicrobial activity, and product application testing have increased. This highlights the growing focus on developing sustainable materials with enhanced functionality such as active properties (antioxidant and antimicrobial activity). Characterization techniques regarding mechanical properties, thermal behavior, and water resistance are crucial for evaluating whether sustainable materials are durable and efficient. Techniques like FTIR, SEM, and TGA predominated, which indicates an emphasis on structural analysis and identification of chemical interactions as well as thermal stability. This is key to ensuring that sustainable materials meet both functional and environmental requirements. Further studies and developments considering real systems, environmental footprint, economic and social assessments are needed toward a broad commercial application of sustainable packages and utensils.

## Supplementary Material





## Notes

Supporting Information 1: Protocol previously registered in the OSF Registries (PDF)
Supporting Information 2: Articles considered in this review (2013–2024) (XLSX)

## Abbreviations

ABTS: 2,2′-Azino-bis­(3-ethylbenzothiazoline-6-sulfonic acid) radical scavenging test
AFM: Atomic Force Microscopy
ASTM: American Society for Testing and Materials
COVID-19: Coronavirus disease 2019
COP21: 21st Conference of the Parties
DPPH test: 1,1-Diphenyl-2-picrylhydrazyl radical scavenging test
DSC: Differential Scanning Calorimetry
DMTA: Dynamic Mechanical Thermal Analysis
FESEM: Field Emission Scanning Electron Microscopy
FTIR: Fourier Transform Infrared Spectroscopy
HDPE: High-density polyethylene
ISO: International Organization for Standardization
LLDPE: Linear low-density polyethylene
LDPE: Low-density polyethylene
NMR: Nuclear Magnetic Resonance
PCL: Polycaprolactone
PBAT: Poly­(butylene adipate-*co*-terephthalate)
PET: Polyethylene terephthalate
PHB: Poly­(3-hydroxybutyrate)
PHBV: Poly­(3-hydroxybutyrate-3-hydroxyvalerate)
PLA: Poly­(lactic acid)
PVOH: Poly­(vinyl alcohol)
SDGs: Sustainable Development Goals
TEM: Transmission Electron Microscopy
TRL: Technology Readiness Level
TGA: Thermogravimetric Analysis
UV–vis: Ultraviolet–Visible Spectroscopy
WVTR: Water Vapor Transmission Rate
XRD: X-ray Diffraction
