# Supplementary material for: Advances in Eco-friendly Materials for Sustainable Packaging and Single-Use Utensils: A Decade of Innovation in Preparation, Characterization, and Applications
Source: ACS Appl Mater Interfaces. 2025 Oct 16;17(43):59032–50. doi: 10.1021/acsami.5c16814 (PMC12581126; doi:10.1021/acsami.5c16814)
Supplement: Supplementary file 1 [file am5c16814_si_001.pdf]

Supporting Information 1

Advances in Eco-Friendly Materials for Sustainable Packaging and Single-use Utensils: A Decade of Innovation in Preparation, Characterization, and Applications

Guilherme Jose Aguilar[1], Alan Maicon de Oliveira[2], Pedro Esteves Duarte Augusto[3], Delia Rita Tapia Blacido[1]*

[1]Department of Chemistry, Faculty of Philosophy, Science and Letters at Ribeirão Preto, University of São Paulo, 14040-901, Ribeirão Preto-SP, Brazil

[2]Ribeirão Preto College of Nursing, University of São Paulo, 14040-901, Ribeirão Preto-SP, Brazil

[3]Université Paris-Saclay, CentraleSupélec, Laboratoire de Génie des Procédés et Matériaux, Centre Européen de Biotechnologie et de Bioéconomie (CEBB), 3 rue des Rouges Terres 51110, Pomacle, France

*Corresponding author: Delia Rita Tapia Blacido (delia@ffclrp.usp.br), Laboratory of Agroindustrial Biopolymer, Department of Chemistry – University of São Paulo, Av. Bandeirantes 3900, CEP 14040-901, Ribeirão Preto, São Paulo, Brazil. Tel.: +55 (16) 33150580

Protocol previously registered in the OSF Registries

REVIEW AIMS:

This study aims to quantify the polymers and key additives used in the production of packaging classified as eco-friendly, sustainable, biobased, and/or biodegradable, as reported in the scientific literature. Furthermore, it seeks to quantify the types of packaging produced and determine the concentration of each additive used.

METHOD:

Design

A scoping review will be conducted based on the methodology developed by the Joanna Briggs Institute (JBI)[1] and aligned with the PRISMA-ScR (Preferred Reporting Items for Systematic Reviews and Meta-Analyses extension for scoping reviews)[2] checklist. This method will be used to address the following guiding question: What are the concentrations of polymers and key additives used in the production of packaging classified as eco-friendly, sustainable, biobased, or biodegradable, as reported in the scientific literature?

Eligibility Criteria

The inclusion and exclusion criteria for the studies will be defined in accordance with the guiding question.

During the screening stages, only experimental and observational studies published between 2014 and May 2024 (the last ten years) and written in English, Spanish, or Portuguese will be considered.

Publications will be excluded if they fall into any of the following categories: review studies, organizational guidelines and recommendations, protocol studies, editorials, lectures, letters to the editor, books, book chapters, and/or conference abstracts.

Search Strategy

The search will be conducted using related descriptors and controlled vocabulary terms (keywords) according to a predefined strategy (Supplementary Material 1) in the scientific database Scopus.

Selection of Evidence Sources and Data Collection Process:

The Rayyan QCRI[3] software will be used to manage the references. All identified references will be imported into the software, and duplicate studies will be removed. Subsequently, two independent reviewers will screen the titles and abstracts of the retrieved studies. In cases of disagreement, a third reviewer will be consulted to resolve the discrepancies.

Next, the reviewers will assess the eligibility of the studies by reviewing the full texts (Figure 1).

A Microsoft Office Excel® spreadsheet will be used for data extraction. The following variables will be collected:

- Quantification of Polymers: Listing and quantification of the polymers identified in the studies;
- Types of Packaging: Classification and quantification of different types of packaging (e.g., bags, containers, films);
- Concentration of Additives: Analysis of additive concentrations in various types of packaging.

Some changes can be made in the data extraction process according to the needs identified during the study.

REFERENCES:

1. Peters MDJ, Godfrey C, McInerney P, Munn Z, Tricco AC, Khalil, H. Chapter 11: Scoping Reviews (2020 version). In: Aromataris E, Munn Z (Editors). JBI Manual for Evidence Synthesis, JBI, 2020. Available from https://synthesismanual.jbi.global. https://doi.org/10.46658/JBIMES-20-12
2. Tricco AC, Lillie E, Zarin W, O'Brien KK, Colquhoun H, Levac D, et al. PRISMA Extension for Scoping Reviews (PRISMA-ScR): Checklist and Explanation. Ann Intern Med. 2018 Oct 2;169(7):467-473. doi: 10.7326/M18-0850.
3. Ouzzani M, Hammady H, Fedorowicz Z, Elmagarmid A. Rayyan-a web and mobile app for systematic reviews. Syst Rev. 2016 Dec 5;5(1):210. doi: 10.1186/s13643-016-0384-4.

SUPPLEMENTARY MATERIAL 1 – SEARCH STRATEGY

1. Scopus

	Search strategy
**#1**	TITLE-ABS-KEY("produc*" OR "prepar*" OR "develop*" OR "applic*" OR "characteriz*" OR "construc*")<br>**AND**
**#2**	TITLE-ABS-KEY("package*" OR "cup" OR "cups" OR "plate" OR "plates" OR "platter" OR "tray" OR "trays" OR "cutlery" OR "knife" OR "knives" OR "spoon" OR "spoons" OR "fork" OR "forks" OR "film" OR "films" OR "foam" OR "foams")<br>**AND**
**#3**	TITLE-ABS-KEY("ecological" OR "sustainable" OR "biobased" OR "biodegradable" OR "biopackag*" OR "bio-packag* OR "bio packag*")
